# Research and Application of Polypropylene Carbonate Composite Materials: A Review

**DOI:** 10.3390/polym14112159

**Published:** 2022-05-26

**Authors:** Xiangrui Li, Lingyu Meng, Yinliang Zhang, Zexiu Qin, Lipeng Meng, Chunfeng Li, Mingli Liu

**Affiliations:** 1 School of Materials Science and Engineering, Beihua University, Jilin City 132013, China; lixiangrui1209@163.com (X.L.); mly578491750@163.com (L.M.); zhangyinliang97@163.com (Y.Z.); q15615125319@163.com (Z.Q.); 2 Jilin Forestry Research Institute, Jilin City 130117, China; menglipengmlp@126.com

**Keywords:** polypropylene carbonate, catalysis, degradation, modification, application

## Abstract

The greenhouse effect and plastic pollution caused by the accumulation of plastics have led to a global concern for environmental protection, as well as the development and application of biodegradable materials. Polypropylene carbonate (PPC) is a biodegradable polymer with the function of “carbon sequestration”, which has the potential to mitigate the greenhouse effect and the plastic crisis. It has the advantages of good ductility, oxygen barrier and biocompatibility. However, the mechanical and thermal properties of PPC are poor, especially the low thermal degradation temperature, which limits its industrial use. In order to overcome this problem, PPC can be modified using environmentally friendly materials, which can also reduce the cost of PPC-based products to a certain extent and enhance their competitiveness in terms of improving their mechanical and thermal properties. In this paper, we present different perspectives on the synthesis, properties, degradation, modification and post-modification applications of PPC. The modification part mainly introduces the influence of inorganic materials, natural polymer materials and degradable polymers on the performance of PPC. It is hoped that this work will serve as a reference for the early promotion of PPC.

## 1. Introduction

**Figure 1:**
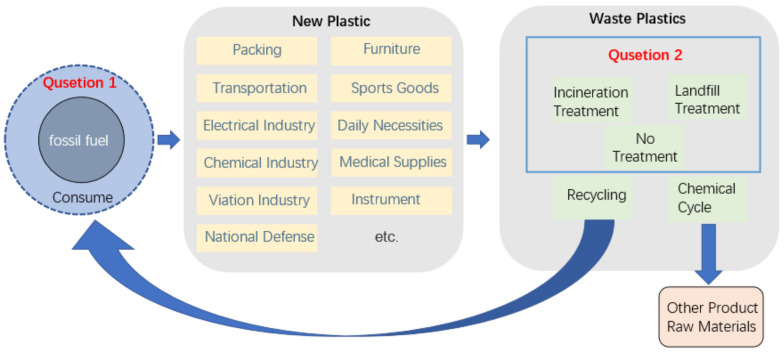
Problems of plastics.

The development and use of plastics has profoundly changed the contemporary world, and plastics have been widely used in packaging, transportation, electronics, chemical, aerospace, national defense, furniture, sporting goods, daily necessities, medical supplies, instrumentation and other fields. Global plastic production reached 348 million tons in 2017 [1]. However, over time, the problems posed by plastics have also been recognized: the accumulation problem and the carbon dioxide (CO_2_) (abbreviations used in this paper are summarized in Table A1) release issues [2]. First of all, the problem directly brought about by plastics is that of accumulation, that is, the problem of plastic pollution. As a polymer material, the properties of plastic are very stable. From manufacturing to complete degradation by nature, the process is very long and can last for decades to hundreds of years [3]. During this process, new plastics are continuously produced, and waste plastics do not fully degrade, resulting in the accumulation of plastics. Four methods are commonly used to treat waste plastics in piles: recycling, chemical recycling, incineration and landfilling [4]. Apart from these methods, the problem of ‘white pollution’ is a direct consequence of discarding waste without any treatment. Among these methods, recycling and chemical recycling both reuse waste plastics, but the reuse rate is very low, resulting in higher costs than producing new plastics and severely limiting their development. Only 9–12% of total global plastics are recycled [5]. Both incineration and landfill methods cause a certain amount of environmental pollution. Plastic pollution can be found everywhere in the habitat of plants and animals and already represents a major threat to their habitat. In addition to plastic pollution visible to the naked eye, microplastics produced during industrial primary production and waste degradation processes have appeared in the living environment through various media, posing a serious threat to human health [6]. Plastic pollution is already an urgent problem for human society. The plastic buildup problem is shown in Figure 1.

On the other hand, a more serious problem is the large amount of greenhouse gases that accompany the production of plastics, which gradually aggravates the greenhouse effect. The main common greenhouse gases are CO_2_, methane (CH_4_), nitrous oxide and fluorinated gases [7,8]. Among the greenhouse gases, there is a need to focus on CO_2_ to mitigate the greenhouse effect. CO_2_ is a common gas in production and life. The combustion of fossil fuels and other industrial activities are often accompanied by CO_2_, which is inevitably produced by human production and life activities [9,10,11]. However, because CO_2_ is a carbon resource, is capture, storage and use are of practical significance with respect to mitigating the effects of CO_2_ on global warming.

Biodegradable plastics have emerged as a response to the problem of plastic accumulation. Degradable plastics can be gradually decomposed into low-molecular-weight polymer chains or small molecules under the stimulus of ozone, water, other molecules, pH, enzymes, mechanical loading and temperature [12]. Biodegradable plastics can be decomposed into water, CO_2_, CH_4_ and other compounds under natural conditions or after treatment [13].

Degradable plastics are mainly divided into bio-based degradable plastics and petroleum-based degradable plastics. Some common biodegradable plastics are thermoplastic starch (TPS), polylactic acid (PLA), polyhydroxyalkanoate (PHA), polyglutamic acid (PGA), etc. Petroleum-based degradable plastics are plastics produced from fossil energy sources, including polybutylene succinate (PBS), polybutylene adipate-co-terephthalate (PBAT), polycaprolactone (PCL), polypropylene carbonate (PPC), etc.

Depending on the degradation mechanism and the form of destruction, they can be divided into fully biodegradable and destructive biodegradable plastics [14]. Completely biodegradable plastics are generally macromolecular structures that can be completely broken down by microorganisms to produce low-molecular-weight, non-polluting inorganic substances, such as PLA, PBS, etc. Destructive biodegradable plastics are degradable plastics made from synthetic materials and natural polymers. Such plastics undergo an oxidation reaction when in contact with microorganisms, causing the molecular chains in the plastic to break and shorten to a point where they can be metabolized by micro-organisms. Examples include cellulose-modified polyethylene (PE), polypropylene (PP), PPC, etc. [15,16,17].

Degradable plastic degradation methods include water degradation, photodegradation, water photodegradation, biodegradation and other methods. With the support of national policies and the enhancement of environmental awareness, the production capacity of degradable plastics has been gradually increased in recent years, and the proportion is increasing in the plastics industry. Common biodegradable plastics are listed in Table 1.

Degradable plastics can alleviate the problem of plastic accumulation to a certain extent, and compared with other degradable plastics, PPC has unique advantages. PPC is a copolymer of CO_2_ and propylene oxide (PO), which can combine CO_2_ as a resource and PO to solve the problems caused by traditional plastics. However, the poor mechanical properties of PPC and the easy degradation of hot processing treatment seriously limit the application and promotion of PPC and PPC-based composites. Therefore, PPC needs to be modified to make up for its shortcomings and to broaden its application areas. It is also necessary to develop and use PPC to adhere to concept of green development.

In this paper, we review the progress of environmental modification of biodegradable material PPC and introduce the synthesis, structure, properties and applications of PPC. Furthermore, the effects of various environmentally friendly reinforcing materials on the performance of PPC mechanical, thermal, degradation and other properties are addressed. The Section 3 focuses on the enhancement of PPC performance by inorganic materials, natural organic polymers and degradable polymers. Based on the performance of PPC and the effect after modification, the specific applications of PPC in several fields are introduced. The main purpose of this review is to facilitate an overall understanding of PPC and to provide guidance on its development, promotion and application.

## 2. Polypropylene Carbonate

Worldwide, the problem of polymer pollution (especially white pollution) caused by improper disposal of waste polymers and inadequate regulations has attracted the attention of various governments [19,20]. These concerns have prompted scientists and industry players to develop and promote biodegradable polymers [21]. PPC is a CO_2_-based, “carbon-sequestering”, fully degradable polymer with great potential for development.

### 2.1. Synthesis of PPC

**Figure 2:**
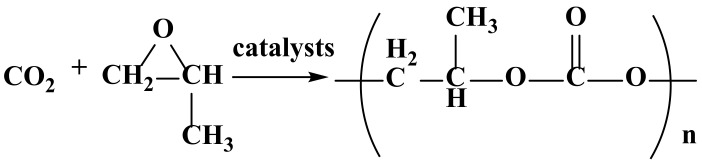
Synthetic reaction of PPC [24].

Inoue first obtained polycarbonate in 1969 from the copolymerization of CO_2_ and epoxide as a raw material, catalyzed by a diethylzinc/water catalytic system [22,23]. This opened the research era of PPC, the synthesis process of which is shown in Figure 2.

In terms of economics, the high price of the product of PO and CO_2_ and the low conversion rate of PPC prepared using a diethylzinc/water catalytic system do not give PPC a great advantage in the polymer market [25]. Hence, some researchers have worked to identify a catalyst with high catalytic efficiency and mild reaction conditions.

The major catalytic systems currently used for the synthesis of aliphatic polycarbonates are zinc-based catalysts, rare earth coordination catalysts, metal porphyrin complex catalysts and bimetallic cyanide catalysts [26,27]. The above catalytic systems can be divided into two main categories: homogeneous and non-homogeneous catalysts. Homogeneous catalyst structures are easily defined and exhibit better activity and product selectivity. Hence, homogeneous catalysts are often more suitable for research. Some non-homogeneous catalysts (heterogeneous catalysts) have shown good production performance in industrial production, although their chemical and crystal structures are not yet clear. Common catalysts and their characteristics are listed in Table 2. Moreover, several commonly used catalysts and their catalytic effects are listed in Table 3. The performance of PPCs produced under different catalytic conditions varies, so a suitable catalytic system needs to be selected according to demand and actual production conditions.

### 2.2. Structure of PPC

**Figure 3:**
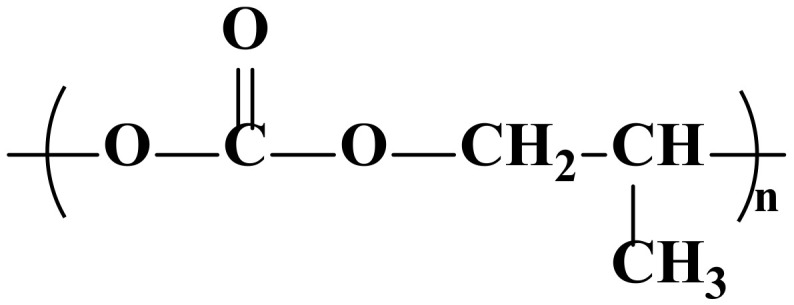
Chemical structure of PPC [49].

PPC is a thermoplastic aliphatic polymer with high barrier properties that can be made from CO_2_ and PO. The use of CO_2_ as a raw material gives PPC a unique “carbon-sequestration” function, and PPC is completely degradable. Its chemical structure is shown in Figure 3.

The presence of ether bonds (-O-) on the main chain of PPC increases the flexibility of the chain and enhances the solubility of the polymer in organic solvents. The presence of the ether bond makes the entire chain segment susceptible to intramolecular rotation around the ether bond, and the molecular chain can easily unravel. The polar carbonyl group (-CO-) increases the intermolecular forces and the rigidity of the molecule. The ester group (-COO-) is easily hydrolyzed and broken, so PPC is susceptible to hydrolysis.

The end group of PPC is a hydroxyl group (-OH), and the thermal stability of PPC is mainly influenced by the end group. At high temperatures, the presence of hydroxyl groups causes lipid alcoholysis, which results in unzipping-type degradation; therefore PPC is thermally unstable. The glass transition temperature (Tg) of PPC is between 30 °C and ~41 °C, i.e., it is not resistant to high temperature.

The side group of PPC is methyl (-CH_3_). The difference in side groups affects the polymer properties. The larger the side group, the more rigid the molecule and the higher the potential resistance, which leads to higher Tg, static flexural strength, melting temperature (T_m_), etc., and lower toughness. However, larger side groups also weaken the intermolecular forces.

### 2.3. Performance of PPC

The properties of PPC are shown in Table 4 on macroscopic level according to the microstructure. PPC has an excellent gas barrier and ductility, which gives PPC potential as a packaging material. The main indicator used to evaluate a material for packaging potential is the gas transmission rate. Table 5 lists the oxygen and water-vapor barrier properties of different polymers. Compared to the oxygen permeability of polyethylene terephthalate, a common reference material for barrier properties, from 0.90 × 10^−13^ to 1.60 × 10^−13^ cm^3^ m m^−2^ s^−1^ Pa^−1^ at 23 °C and pure PPC exhibited OPs of 4.16 × 10^−13^ to 8.71 × 10^−13^ cm^3^mm^−2^s^−1^Pa^−1^ [50]. Compared with other polymer films, PPC has better oxygen barrier properties, so iy is often used in low-temperature packaging materials. Due to this property, in practical production applications, it is usually necessary to modify PPC to enhance its comprehensive performance [51].

Mechanical properties are an important and fundamental function in the evaluation of materials. The main indicators of the mechanical properties of materials are modulus of elasticity, strength at break, yield strength, elongation at break, tensile strength, etc. As PPC is an amorphous polymer with low intermolecular forces, its mechanical properties are poor [52]. It has been shown that the mechanical properties of PPC are influenced by the Tg and molecular weight [53]. For a PPC with a Tg of 40.3 °C, the energy storage modulus drops from 1000 MPa to 10 MPa as the temperature rises from below Tg to the glass transition zone. As the number-average molecular weight of PPC increased from 109 kg/mol to 227 kg/mol, its modulus increased at both temperatures above and below the Tg [54]. The thermal properties of PPC and the reasons for the poor thermal properties were described in the Section 2.2 and will not be repeated here.

Additionally, PPC has good melt processability and biocompatibility, which provide a variety of possibilities for PPC modification, will be described specifically in Section 3.

### 2.4. The Cyclicality and Sustainability of PPC

**Figure 4:**
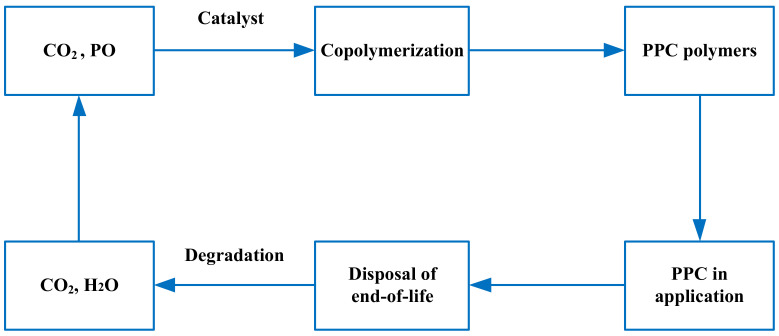
Life cycle of PPC.

The raw materials for PPC production are CO_2_ and PO, of which CO_2_ is widely used and cheap. Although PO is slightly more expensive, PPC can still offer significant economic benefits. In the process of catalytic synthesis mentioned above, there is no lack of synthesis methods for PPC at room temperature and low pressure, which reduces the synthesis difficulty and processing cost of PPC to a certain extent. During the production of PPC, no new harmful substances are produced, and CO_2_ is fixed in the PPC. PPC will degrades to CO_2_ and other non-polluting substances. This means that no new CO_2_ is generated throughout the production and utilization of PPC until its degradation. Combined with CO_2_ capture and fixation technology, a certain amount of CO_2_ can be dynamically recycled to meet production needs and mitigate the environmental crisis caused by the greenhouse effect. The life cycle of PPC is shown in Figure 4.

As mentioned above, PPC is a recyclable and degradable green polymer. The ability to “sequester carbon” from CO_2_ is a unique advantage of PPC over other biodegradable polymers. On the one hand, it saves resources, and on the other hand, it fixes CO_2_, which reduces greenhouse gas emissions to a certain extent. These factors are critical to sustainable development and to addressing plastic buildup and the greenhouse effect.

### 2.5. Degradation of PPC

Complete degradation is one of the advantages of PPC, which can be degraded in various ways, such as thermal degradation, biodegradation and hydrolysis (under acid and alkaline conditions). The effects of biodegradation and hydrolysis are mainly after the PPC has been put into use, causing the PPC to gradually age and lose its performance. Biodegradation and hydrolysis are the advantages of non-polluting degradation of PPC. However, biodegradation and hydrolysis also make PPC products susceptible to unwanted aging and other problems caused by environmental influences during use. The problem affecting the production of PPC is thermal degradation. The decomposition onset temperature of PPC is low, ranging from 180 °C to 240 °C. In industrial production, the processing temperature is often above 200 °C, which can cause certain degradation of PPC during thermal processing and affect the performance of PPC materials.

**Figure 5:**
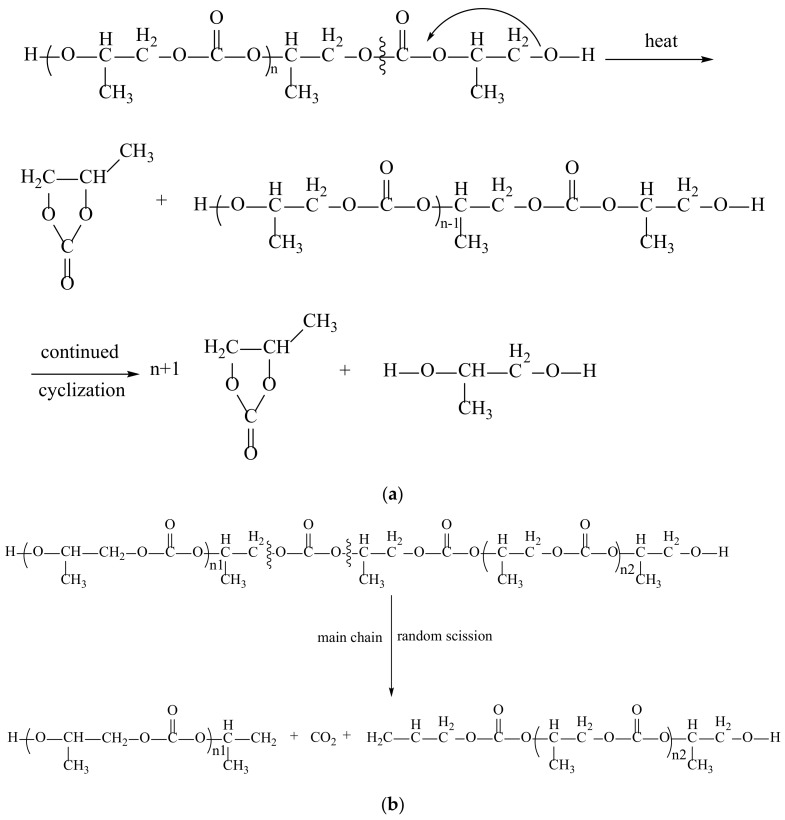
Thermal degradation of PPC. (**a**) Unzipping degradation; (**b**) unconventional chain-breaking degradation [60].

Shouhei Inoue proposed a mechanism for thermal degradation of PPC in 1975. After subsequent specific studies, the thermal degradation of PPC was determined as random chain-breaking degradation and “back-bite”/unzipping degradation [56,57]. It was subsequently pointed out that PPC is susceptible to unzipping degradation at lower temperatures and random chain-breaking degradation at higher temperatures. Among them, the main factor affecting the unzipping decomposition of PPC is the terminal hydroxyl group, and capping the hydroxyl group is an effective method to inhibit zipper degradation. However, the seal-end method, cannot solve random chain-breaking degradation, although the use of some organic additives can effectively inhibit the random chain-breaking degradation. The unzipping degradation mechanism is shown in Figure 5a. The random-break degradation mechanism is shown in Figure 5b. In this case, the product of PPC unzipping decomposition is cyclic carbonate, and random-break degradation produces CO_2_ and generates compounds containing terminal alkene bonds [58]. Although no polluting substances are produced, thermal degradation affects the performance of PPC to some extent, so this is a major problem faced by PPC production applications. After decatalyzation of PPC, its degradation rate was found to lower than that of PPC without decatalyzation, even when the degradation temperature was reached [59]. However, PPC cannot be prepared without the use of catalysts, and there will always be some catalyst residues in PPC, so it is necessary to seek suitable catalysts, refined PPC removal or other methods to enhance the performance of PPC.

**Figure 6:**
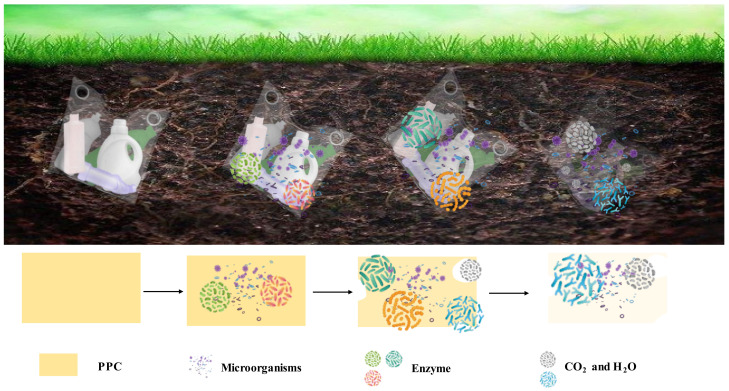
Biodegradation of plastics in natural environments [65,66].

Biodegradation is an advantage of PPC [61]. There are various types of microorganisms and biodegradable flora in the soil, and when PPC or modified PPC is placed directly in the soil environment, microorganisms start to erode from its surface, which leads to degradation by splitting, oxidation and chain breaking [62]. A degradation schematic is shown in Figure 6. Some studies have shown that the degradation of PPC membranes placed in soil extracts is poor, probably due to the hydrophobic nature of PPC itself, which prevents micro-organisms from growing on its surface, leading to unsatisfactory degradation rates [63]. Compared the degradation behavior of PPC placed in a soil environment, the degradation of PPC in a soil extraction buffer was faster, probably because the products of degradation were washed away and stripped by the buffer, allowing the degradation to continue [64]. We do not want micro-organisms to corrode PPC materials during production, so in practical application, certain anticorrosion treatments of PPC materials or PPC-based materials can extend their service life.

There are other factors that can affect the degradation of PPC, including light, acidity and alkalinity. Sang Liangyong and colleagues [67,68,69] investigated the effects of light, alkaline conditions and seawater environment on the degradation behavior of pure PPC, as well as PPC/PLA copolymers. In conditions of simulated light from a xenon lamp, pure PPC is susceptible to degradation due to the softness of the PPC molecular chains. After PPC has been blended with PLA, light may cause the blends to produce free radicals, which further interact with certain groups or atoms to promote photodegradation. As a result, the PPC/PLA blend loses more mass under xenon light conditions than pure PPC. Under seawater conditions, the ester bonds of PPC are prone to hydrolysis and breakage, so that after 240 days of degradation in seawater, PPC in its pure form develops a large number of tiny holes. The PPC and PLA in the 50/50 PPC/PLA blend are partially compatible, and there is a certain interaction between the two that makes it difficult for water molecules to react with the ester bonds; therefore the degradation of the blend is lower than that of pure PPC. PPC degrades when placed in an environment of NaOH solution to produce acidic substances, such as carboxyl groups, which further contribute to the positive reaction of hydrolysis. Furthermore, in the degradation of PPC, the quality and strength of the plastic usually decrease with time, although degradation of the plastic during normal use is not expected. It would be misleading to measure PPC degradation when it is not uniformly degraded. Yang Xuxu and colleagues [70] explored the non-uniform degradation of extreme forms of degradable plastics and found that the cracking rate was insensitive to load and sensitive to relative humidity and pH. This is a guideline for the study of PPC degradation.

It is necessary to consider the influence of various factors on the degradation of PPC in different environments. The advantage of is that the degradation rate is fast in a specific environment, not causing problems of “microplastics” and “plastic accumulation” brought about by the degradation of petroleum-based plastics and not producing new CO_2_.

## 3. Modification of PPC

**Figure 7:**
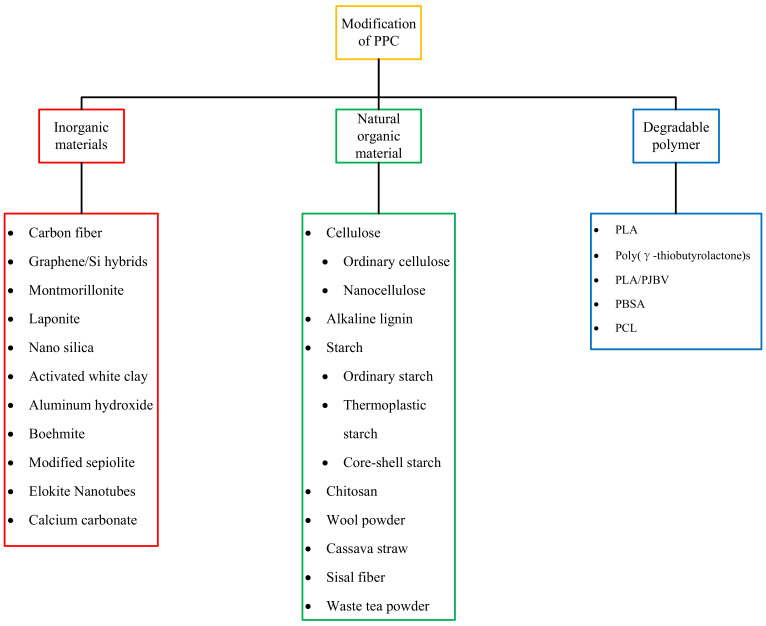
Some materials that can be used for PPC modification.

The thermal stability of PPC prepared from homogeneous catalysts may be compromised during the preparation process due to its poor thermal stability, which is produced at around 230 °C in industrial production of normal industrial polymers. Moreover, due to the poor mechanical properties of PPC, the above defects seriously limit its application in industrial production. Therefore, PPC needs to be modified to improve its performance and expand its industrial applications. In order to broaden the field of application of PPC, other materials are usually selected to be fused with PPC to prepare composite materials. The main focus here is on the modification of PPC with green materials (inorganic materials, natural organic polymers, biodegradable polymers, etc.). The use of such degradable materials allows the modified PPC to retain its degradability, which is still in line with the green development route. The modified materials introduced in this paper are shown in Figure 7.

The main common blending methods are melt blending and solution blending. Melt blending is a common method of modifying many thermoplastics and is technically mature. Melt blending comprises the use of thermoplastics to achieve a molten state when blended with other materials. Melt blending is industrially easy to implement, simple to operate and does not require solvent recovery operations. Solution blending is a more complex procedure whereby PPC is mixed with other materials in a solvent state, resulting in a more complete blend. Both melt blending and solution blending methods require a second substance or substances to blend with PPC to improve the properties of the composite.

### 3.1. Inorganic Material Modification

Inorganic materials are diverse, abundant and inexpensive, and mineral materials are often used as filler materials to enhance the mechanical and thermal properties of other materials. Moreover, a variety of inorganic mineral materials have special properties that can often contribute a certain functionality to the material. The main inorganic materials presented here are carbon fibers, graphene oxide, laponite, montmorillonite (MMT), modified silica nanoparticles, activated white clay (AC), aluminum hydroxide (ATH), elokite nanotubes (HNTs), calcium carbonate and other materials. Furthermore, different processing methods and additives are used in the preparation of composites. The inorganic materials used to modify PPC and their modification effects are shown in Table 6.

### 3.2. Modification of Natural Organic Polymer Materials

Modification of PPC using polymeric materials, especially natural ones, often achieves better results [81,82]. In this regard, natural degradable polymers, such as cellulose, lignin, starch, chitosan (CS) and wool, are renewable, degradable, widely available and inexpensive [83]. Because natural polymers are inherently biodegradable, the composites produced by mixing PPC with natural polymers are still biodegradable. The complete degradability of natural polymer-modified PPC does not put pressure on the ecological environment.

Of the natural polymers, cellulose, the main component of the cell walls of plants, is simple and easy to obtain and is the natural polymer with the largest reserves. The twin-screw extruder method is commonly used for melt blending; therefore, the melt blending process of PPC/cellulose composites can be achieved with the help of a twin-screw extruder. The addition of cellulose enhances the tensile properties and energy storage modulus of the composite. However, the compatibility between PPC and cellulose is poor, and the reinforcing effect is somewhat limited. The addition of cellulose hinders the molecular-chain movement of PPC to some extent, which can enhance the thermal stability and Tg of composites [84].

Once a material reaches the nanoscale, some superior functionality often emerges. Nanocellulose (NCC) has more excellent properties than natural cellulose, such as high specific surface area, high crystallinity, high strength and ultra-fine structure [85]. Some studies have shown that the tensile strength of PPC increases with the increase in NCC content when the NCC addition does not exceed 1.5%. As the NCC content exceeds 1.5%, agglomeration of NCC occurs, which leads to a decrease in the tensile strength of PCC. The composite material changes from ductile fracture to brittle fracture.

Lignin is also a common natural polymer, and byproducts from the paper industry are often include alkaline lignin (AL), which is difficult to utilize. Lignin is a renewable, degradable and heat-resistant material with a Tg of between 100 and 180 °C. A considerable amount of research has been carried out to make effective use of lignin, not least of which is the preparation of composites from AL mixed with PPC. PPC/AL composites can also be prepared using melt-blending methods, and for composite studies the blending ratio is the first issue of concern. The thermal and mechanical properties of PPC/AL composites were investigated at 10% and 40% AL content [86]. The Tg of PPC/AL composites was found to be 30.9 °C at 10% AL content, which was 8.9 °C higher than that of pure PPC; the tensile strength of PPC/AL composites was 13.44 MPa at 40% AL content, which was 213% higher than that of PPC, and the elongation at break was 115%. However, AL is poorly dispersed, and therefore, modification is often required. Chemical modification of AL using formaldehyde enhances the dispersibility of the lignin. Formaldehyde-modified black liquor lignin (BLF) is then blended with PPC using a melt-blending method. The tensile strength, modulus of elasticity, thermal stability and processing stability of composites were significantly improved after the addition of small amounts of BLF due to its good dispersion [87].

BLF could also be modified with hydroxypropylation to make modified hydroxy black liquor lignin (HBL) [88]. The unmodified lignin is prone to agglomeration, whereas HBL has a lower thermal transition temperature and higher melt flow, resulting in better dispersion of HBL in the PPC matrix. As a result, the tensile properties, thermal stability and processing stability of the PPC/HBL composites are improved. In addition, the hydrophilicity of the composites is improved, which accelerates the natural degradation rate of the composites.

Starch is a natural polymer that is completely degradable and has the advantages of being non-toxic and inexpensive. Starch can be melted and blended with PPC to enhance its performance. However, there are two problems with this treatment method. First, starch is a polysaccharide polymer compound with a large number of hydroxyl groups in its molecules, which can form a large number of intra- and intermolecular hydrogen bonds and complete granules with a microcrystalline structure, resulting in a Tm much higher than the degradation temperature. As a result, common starch does is not thermoplastically processable. PPC is also less compatible with starch, which leads to limited modification. To overcome these problems, there are usually two solutions. On the one hand, reactive bulking agents, such as succinic anhydride, diphenylmethane diisocyanate, maleic anhydride, etc., can be added to the blend of PPC and starch. On the other hand, starch can be pretreated by grafting and esterification before being blended with PPC to prepare composites. It has been shown that oxidation of starch can improve compatibility with biodegradable materials [89,90].

In the first type of solution, one step of using maleic anhydride as a reaction capacitor is as follows. PPC was first grafted to prepare maleic-anhydride-grafted PPC (PPCMA), and then PPCMA was melt-blended with TPS, thermoplastic starch oxide (TPOS) and aluminate-pretreated starch oxide (DL-TPOS) to compare the modification effect of each of the three [91,92]. Hydrogen bonding is formed between PPCMA and starch, so the tensile strength of PPCMA/TPS composites is increased compared to that of pure PPC. For PPCMA/TPOS composites, the partial conversion of hydroxyl groups in TPOS to carbonyl groups enhances the compatibility between PPCMA and TPOS, and the PPCMA/TPOS composites have improved tensile and impact strength, energy storage modulus, loss modulus and complex viscosity. DL-TPOS reduces the exposure of hydroxyl groups on the starch surface, reduces the probability of starch granule agglomeration, improves dispersion and further enhances the tensile strength of the composite. At a DL-TPOS content of 40%, the tensile strength of a PPC/DL-TPOS composite was increased by 460% compared to pure PPC. A maximum value of 13.72 MPa was reached.

The presence of a large number of hydrogen bonds between starch molecules is not conducive to melting operations. PPC is hydrophobic, whereas common starch is hydrophilic and rigid, resulting in poor compatibility between the two materials. Core-shell starch nanoparticles (CSS NPs) can be used as the core, and the shell is prepared using a poly (methyl acrylate) (PMA), which is produced in the soap-free emulsion copolymerization of methyl acrylate. The combination of the flexibility of PMA shells and the flexibility of CSS NPs resulted in improved compatibility of microcapsules with PPC, which significantly enhanced the mechanical and thermal properties of the PPC/CSS composites. The improved compatibility of starch with PPC, which has a core-shell structure, enhanced the mechanical and thermal properties of the PPC/CSS composite [93].

CS is the second most abundant natural organic polymer after cellulose. In the PPC/CS composites, the compatibility, Tg, thermal weight, loss temperature and tensile properties of the PPC/CS blends were examined for different levels of CS, and the effect of different levels of CS on the abovementioned properties of the PPC/CS blends was analyzed [94]. The tensile properties of PPC/CS increased when CS content was increased to 20%, and the tensile properties of PPC/CS composites increased from 4.7 MPa to 12.5 MPa when the CS content reached 20%. Moreover, the addition of CS also inhibited unzipping-type degradation in the low-temperature region of PPC and improved the heat resistance of PPC. O-lauroyl chitosan and PPC are blended using the solution casting method [95]. Hydrogen bonding interactions are formed between PPC and OCS. The tensile properties, elongation at break and Young’s modulus of the PPC/OCS blended film increase.

Wool is a widely used animal fiber, and its specific surface area is significantly increased when it is ground into granules to make wool powder (WP). WP can be used as an effective additive to modify PPC, and the thermal and mechanical properties of PPC can be significantly improved by blending PPC with WP using the solution-blending method and making a film by stirring and drying [96].

Besides the abovementioned common natural polymers, many researchers have blended agricultural residues containing the above components with PPC, such as straw [97], waste tea leaf powder [98], sisal [99], eggshell powder [100] and waste wood powder.

### 3.3. Degradable Polymer Modification

Other biodegradable plastics, such as PLA, β-hydroxyvalerate copolymer (PHBV) and PCL, are also often used to modify PPC. As both PPC and PLA are environmentally friendly materials, the blending of the two can provide complementary advantages, although their poor compatibility has led to much research [101,102]. Integral to this type of research is the study of the effect of blending ratios on the performance of PPC/PLA composites. The effect of variations in PPC content on the overall properties of the composite was analyzed under melt-blending preparation conditions. As the PPC content of the blend increases, the flexibility of the molecular arrangement increases and the elongation at break of the PPC/PLA blend increases significantly, with a relative decrease in tensile strength. Thermal properties analysis, fracture surface analysis and Fourier transform infrared (FTIR) spectroscopy analysis yielded a partially blended phase of PPC and PLA. Analysis by differential scanning calorimetry showed that the Tg of PLA decreased as the PPC content of the blend increased [103]. Similar conclusions were reached in a study by Wang Shufang et al. [104]. In PPC/PLA blends, a modifier, long-chain hyperbranched polymer (LCHBP), can be added to improve PLA/PPC compatibility, as PLA/PPC are partially compatible. The modification of PLA/PPC blends using LCHBP yielded a much higher elongation at break and impact strength of the blends, whereas the tensile strength was essentially unchanged, and the brittleness of the modified blends was increased [105]. Besides simply studying the blending of two materials, PPC and PLA, some inorganic minerals and degradable polymers can be used to prepare ternary blends in order to retain their degradability. PLA/PPC-modified montmorillonite (OMMT) nanocomposites can be prepared by adding organic OMMT to the PLA/PPC system using a twin-screw extruder [106]. When the ratio of PLA/PPC was 80/20 and the content of OMMT was 1.5%, the tensile strength, elongation at break and impact strength of the composites reached maximum values of 51.07 MPa, 3.53% and 13.38 kJ/m^2^, respectively.

Three environmentally friendly materials, PLA, PPC and PHBV, can be used to prepare PLA/PPC/PHBV composites using the solution-casting method [107]. Different ratios (60/20/20, 40/20/40, 40/40/20, 20/60/20, 20/40/40 and 20/20/60) were chosen to prepare the blended films. The Tg of the blended system varies with the content of the three components. At constant PLA and PPC content, the Tg of the blends decreases with increasing PHBV content, and the opposite is true for PLA. When the PLA content of the blend increases, the Tg of the blend rises. In addition, the Tm of the blended system is generally lower than the Tm of the monomer. Such a ternary blending system allows the three materials to complement one another, with PLA, PPC and PHBV improving the strength, elongation at break and biodegradation rate, respectively. Blending PBSA with PPC can also enhance the thermal and mechanical properties of PPC [108].

Biomass-derived degradable poly(γ-thiobutyrolactone)s are new materials that have recently been developed with high thermal and mechanical properties. More notably, poly(γ-thiobutyrolactone)s can be ultra-rapidly degraded (within 15s) to the parent γ-thiobutyrolactone by the catalytic action of commercially available 1,5,7-triazabicyclo[4.4.0]dec-5-ene organocatalyst (0.2 mol%) thiobutyrolactone. This material is recyclable in a real sense [109]. The coblending modification of PPC with poly(γ-thiobutyrolactone)s might be a feasible solution.

In the case of poor compatibility between the blends, it may also be possible to improve the compatibility between the blends by first modifying one or all of the blends. In the PPC/PCL blending system, Si-g-PCL composite nanoparticles were prepared by introducing C-OH on the surface of Si nanoparticles, using a melt-blending method as a means of modifying PPC [110]. The mechanical properties and thermal stability of the material were eventually enhanced. Enzymatic degradation experiments have also demonstrated that Si nanoparticles and Si-g-PCL composite nanoparticles improve the enzymatic degradation of PPC without compromising biodegradability.

### 3.4. Other Modification Methods

Modification of PPC by chemical reagents is also a common method. Although physical modifications are easy to achieve industrially, they have a limited range of modifications to adjust, and the modified PPC properties obtained from chemical modifications tend to be more stable. Chemical modification requires the introduction of appropriate units into the polymer, starting from the microstructure, so that a chemical reaction can take place to copolymerize the molecular chain structure [111,112]. Chemical modification methods include terpolymerization, capping, cross linking, chain transfer, block copolymerization and graft copolymerization [113,114,115]. Chemical reagents may affect the environmental friendliness of PPC and are therefore not described in this paper.

In summary, the modifications carried out on PPC are based on retaining the advantages of PPC biodegradation and improving the defects of PPC by various means.

## 4. Applications of PPC

PPC has good macroscopic properties, such as non-toxicity, gas barrier (oxygen barrier), biodegradability [116], biocompatibility [117], toughness and low-temperature flexibility. These properties have led to the use of modified PPC in a wide range of applications, and the green material modification of PPC has not only retained its inherent ability to “sequester carbon” but has also overcome its poor mechanical and thermal properties and broadened the application areas of PPC.

**Figure 8:**
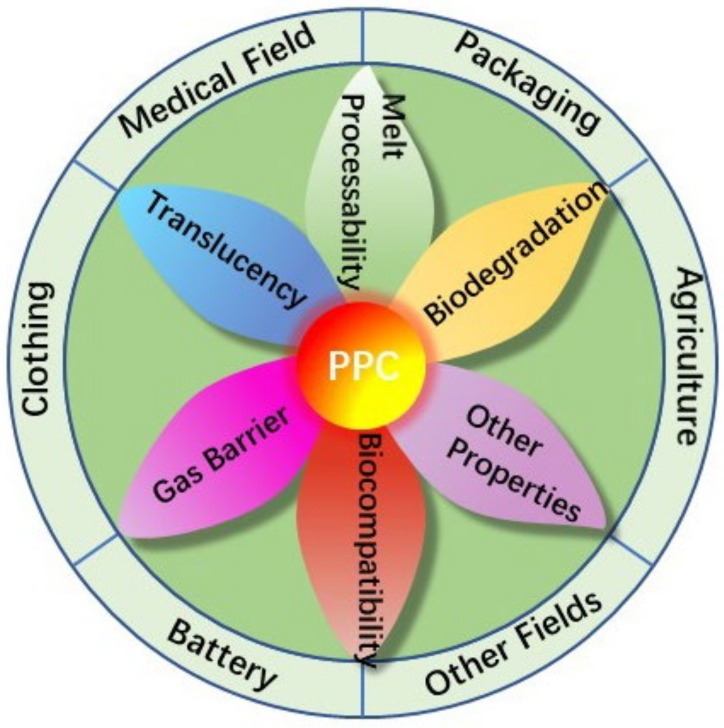
Application areas of PPC. (Style referenced from [122]).

PPC has a wide range of applications, and due to its widely exhibited properties, it is now widely used in film-like materials, cushioning foams [118], adhesives, medical materials [119], battery materials [120], sheets, plastic modification aids, toughening agents, coatings/inks and many other fields. It has the potential to replace PE and PP in the preparation of disposable materials [121]. The application areas of PPC are shown in Figure 8.

PPC can be used in the textile industry for non-woven fabrics. Biodegradable PPC-based composite spunbond non-wovens were prepared using a twin-screw extruder and an integrated spinning machine to melt-blend, draft and hot-press PPC with PP as an additive [123]. Environmentally friendly spunbond non-woven slices were prepared using PLA and PPC, whereby PLA and PPC were capped by accessing maleic anhydride during the melting process; the prepared materials met the process requirements for spunbond non-woven fiber forming [124]. Non-wovens technology has been applied to a wide range of plastics, and treating waste PPC for such applications is one way to reuse PPC.

Because PPC has good transparency and high barrier properties and is completely degradable, some researchers have prepared it as a floor membrane [125]. PPC is degraded by micro-organisms in the soil environment. Although degraded PPC increases the pH of soil, it does not significantly affect the richness of the microbial community in the soil [126]. Nevertheless, PPC degrades slowly under normal conditions and does not meet usage requirements. Cellulose acetate (CA) is a widely used class of thermoplastic resin membrane materials; the presence of ester groups in CA allows for improved compatibility with polyester-based polymers. Therefore, Cai Zhuo et al. [127] added CA to PPC to prepare a composite film with better biodegradability, and the mechanical properties and thermal stability of the composite film were also significantly improved. This kind of laminated film is more in line with real-life production applications. PPC and PCL can be used to prepare a PCL/PPC blended film, and unlike mulch films, PCL/PPC blended films can also be used for food preservation of mushrooms. This requires the co-blended membrane to have good gas-barrier and water-vapor permeability. PCL/PPC50 met these requirements, and no condensation occurred in the composite. This composite also has a special function in preserving polyphenol oxidase activity and mushroom color [128].

As a packaging material, especially for medical supplies or food, it is necessary to maintain good antibacterial properties and a long time to maintain antibacterial properties. Traditional antimicrobial treatments include the application of antimicrobial coatings to the surface of packaging materials or the use of nanoparticles, organic acids and antimicrobial polymers to create antimicrobial packaging materials. Both of these methods may have a negative impact on the environment, as well as the packaged material. Such problems can be avoided by using plasma for the treatment of packaging materials. If the natural antimicrobial compound thymol is first immobilized on the PPC surface using plasma technology, its maximum antimicrobial activity is retained. The antimicrobial activity of modified PPC was maintained for 7 days and several months under aqueous media conditions and dry conditions, respectively [129].

Because of its good biocompatibility, PPC was placed under the surface of experimental mice and exhibited good biocompatibility, with no extensive chronic inflammation or tissue necrosis at the implanted site. PPC instead underwent degradation behavior within the organism [130]. Therefore, blending of hydroxyapatite (HAP), which is often compounded with other biomedical materials, such as PLA, PCL, PLGA [131] and PHA [132], with PPC was attempted. As HAP is one of the components of human bone, it has good biocompatibility and osteoconductivity. The research team tried to investigate the effect of the addition of HAP on the performance of PPC. The experimental results showed that the addition of HAP gradually changed the fracture of PPC from ductile fracture to brittle fracture; the fracture strength, impact strength and hydrophilicity of PPC/HAP composites increased with increased HAP content. The elongation at break of the composites decreases [133]. The improvement of the above properties offers the possibility of PPC as a medical material.

In the field of medical dressings, PPC/diatomaceous earth medical composite fibrous films can be prepared using electrostatic spinning technology [134]. The best process for preparing composite membranes was determined by orthogonal tests: the mass fraction of spinning solution was 7%, the mass fraction of diatomaceous earth was 0.8%, the inner diameter of the needle was 0.6 mm and the receiving speed was 60 r/min. A bacteria inhibition test found that when the mass fraction of diatomaceous earth is 0.8%, the composite fiber film has a certain degree of bacterial inhibition up to 69.3%. Therefore, PPC/diatomaceous earth composite fiber film can be used for medical dressing.

In the field of dentistry, caries disease is a common chronic infectious disease. Fluoride is considered to have the potential to prevent and treat dental caries. However, the duration of fluorine residence in the oral cavity affects the effectiveness of treatment of dental caries. Fluoride-based PPC with good caries prevention action was prepared by melting and blending PPC with fluorine [135].

The demand for lithium-ion batteries with high energy density and long service life is expanding in today’s society. However, the poor thermal stability of lithium-ion batteries at high temperatures requires a focus on the development of solid-state electrolytes. Solid electrolytes for lithium-ion batteries comprise two types: inorganic solid electrolytes and polymer electrolytes. PPC can be used as a polymer electrolyte with excellent performance. The excellent performance of PPC-based electrolytes includes higher ionic conductivity, wider electrochemical window and excellent thermal stability. The use of the ionic liquid 1-vinylimidazole bis(trifluoromethanesulfonyl)imide (VIm-TFSI) plasticizer incorporated into a PPC matrix significantly enhanced the ionic conductivity of the PPC-based electrolyte and improved the interfacial similarity between the PPC-based electrolyte and the electrode [136].

All-solid-state lithium batteries can be prepared using PPC electrolytes with LiNi_0.5_Co_0.2_Mn_0.3_O_2_; however, an oxidation reaction occurs between LiNi_0.5_Co_0.2_Mn_0.3_O_2_ and the PPC-based electrolyte, and Ni^3+^ and Co^4+^ can decompose the PPC into aldehydes. However, graphene was used as a sandwich layer to solve this problem [137]. The introduction of graphene interlayers onto the surface of LiNi_0.5_Co_0.2_Mn_0.3_O_2_ cathodes can attenuate side reactions and enhance the structure of the cathode, improving the electrochemical performance of solid-state lithium batteries. In addition, PPC can be used in the bonding process of PPC-based aluminum-coated silicon wafers [138].

Nanocomposites with shape-memory PPC have also been applied in smart materials [139,140]. In addition, in order to cope with the current shortage of wood materials, PPC can be used as a supplementary material to replace some wood materials to prepare PPC-based wood–plastic composites with better performance [141].

## 5. Conclusions

PPC is a fully degradable and “carbon-sequestering” material that can cope with the problems of plastic accumulation and the “greenhouse effect”. Many studies have been conducted to modify PPC by physical or chemical means to address its thermal and mechanical properties and other deficiencies, resulting in modified PPC composites with improved properties. In this paper, we reviewed the synthesis, catalysis, modification and degradation of PPC, as well as the effect of various inorganic materials, natural organic polymers and degradable polymers on PPC modification. The inherent ability of PPC to “sequester carbon” makes it very suitable for today’s green development. On the basis of its modification, PPC will play a more important role in many fields. This article will hopefully provide a clear understanding of PPC for those involved.

## Figures and Tables

**Table 1:** Summary of common biodegradable plastics [18,19].

Type	Abbreviation	Structural Formula	Application
Thermoplastic starch	TPS	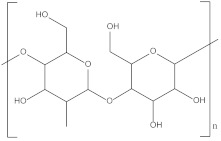	Packaging, shopping bags, garbage bags, mulch films, disposable tableware and disposable medical products.
Polyhydroxyalkanoate	PHA	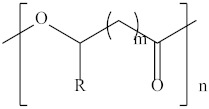	Tissue engineering, medical implants, controlled drug delivery systems, packaging, mulch films and disposable medical products.
Polyglutamic acid	PGA	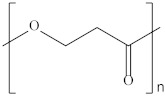	Food thickeners, stabilisers, surgery, food, cosmetics, pharmaceutical industry, agriculture.
Poly (lactic acid)	PLA	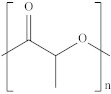	Packaging, shopping bags, garbage bags, mulch films, disposable tableware, disposable medical products, building materials and textiles.
Poly (butylene succinate)	PBS	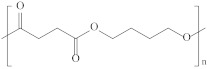	Packaging, shopping bags, garbage bags, pesticide and fertilizer sustained-release materials, mulch films and disposable tableware.
Poly (butylene succinate-co-butylene adipate)	PBSA	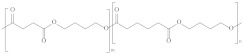	Packaging, shopping bags, garbage bags and mulch films.
Poly (butylene adipate-co-terephthalate)	PBAT	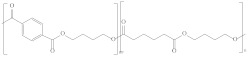	Packaging, shopping bags, garbage bags, mulch films and disposable tableware.
Polycaprolactone	PCL	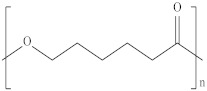	Medical implants, controlled drug delivery systems, absorbable surgical sutures and cryogenic packaging.
Poly (propylene carbonate)	PPC	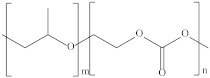	Cryogenic packaging, mulch films, foam materials, controlled drug delivery systems and high-barrier materials.
Poly (vinyl alcohol)	PVA	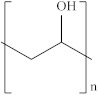	Soluble packaging, high-barrier materials and medical implant.

**Table 2:** Catalysts for the copolymerization of CO_2_ and epoxides [28,29].

Category	Typical Catalyst		Features
Heterogeneous catalysts	ZnEt_2_-active hydrogen [25,30,31]	(1)	Low catalytic activity.
		(2)	High price.
	Zinc carboxylic acid [32]	(1)	Easy preparation and low cost.
		(2)	Long reaction time.
	Double metal cyanide complex [33,34]	(1)	High catalytic activity.
		(2)	Polymers with low Mn ^(a)^ and low CO_2_ fixation.
	Ternary rare-earth catalyst [35]	(1)	Mn over 100 kg/mol in a relatively short time.
		(2)	Catalytic activity needs improvement.
Homogeneous catalyst	Metal-porphyrin [36]	(1)	High catalytic activity but very slow polymerization rate.
		(2)	Catalyst structure is clear.
		(3)	Simple to synthesize and easy to handle.
		(4)	Product may have an undesirable color.
	Zinc and cadmium phenoxides [37,38]	(1)	Catalyst structure is clear.
		(2)	Rapidly induced copolymerization.
		(3)	Most of the polymers have a molecular weight of less than 100 kg/mol.
	β-Diiminate zinc [39,40]	(1)	Catalyst structure is clear.
		(2)	Controlled ring opening.
	Metal-salen or -salan complexes [41]	(1)	Catalyst structure is clear.
		(2)	High selectivity.
		(3)	High catalytic activity.
		(4)	Product may have an undesirable color.

^(a)^ Mn: number-average molecular weight.

**Table 3:** Catalytic effect of different catalysts.

No.	Catalyst	PPC Yield ^(a)^	PPC Product				Ref.
			Mn ^(b)^	Mw ^(c)^	PDI ^(d)^	[η] ^(e)^,dL/g	
1	ZnGA	83 (g polymer/g of catalyst)	160 k	60 k	2.7	-	[42]
2	ZnGA + GA	68.25 (g polymer/g of catalyst)	-	-	1.2815	-	[43]
3	-RE(P_204_)_3_-Al(i-Bu)_3_-R(OH)n	1672 (g/mol of Y ^(f)^)	46.9 × 10^−4^ (g/mol)	-	-	3.82	[44]
4	Nd(CCl_3_COO)_3_-ZnEt_2_-glycerol ternary catalyst	Improving	62,282	73,412	-	0.76	[45]
5	Lewis Base	416.1 (g/(mol Zn))	11.0 × 10^−4^ (g/mol)	-	2.9	-	[46]
6	Zn_3_ [Co(CN)_6_]_2_-based Co-Zn DMC catalyst	7488 (g polymer/g of catalyst)	35,900	-	3.99	-	[47]
7	Zn-Mg-Al composite oxide high-efficiency catalyst	88.8%	-	-	-	-	[48]

^(a)^ Because the units used are not uniform across studies, the units here are different. ^(b)^ M_n_: number-average molecular weight. ^(c)^ M_w_: weight-average molecular mass. ^(d)^ PDI: polydispersity index. ^(e)^ [η]: intrinsic viscosity. ^(f)^ Y: yttrium.

**Table 4:** Performance of PPC [55].

Characteristic	Numerical Values
Glass transition temperature (°C)	30, 33, 41
Elastic modulus (MPa)	993
Tensile strength (MPa)	33.2
Density (10^3^ kJ/kg)	1.275, 1.3
Permittivity (kHz)	3.0
Combustion heat (10^3^ kJ/kg)	18.5
Refractive index, n	1.463
Hydroscopicity (23 °C, %)	0.397
Thermally decomposed temperature (°C)	218
Venting quality N_2_ (ml-cm10^−12^)	5.3

**Table 5:** Barrier properties of different polymers [30].

Material	H_2_O (g/m^2^/24h)	O_2_ (cm^3^/m^2^/d/atm)
PPC	40–60	10–20
Biaxially oriented polyethylene terephthalate	100	60–100
Bidirectional oriented polypropylene	-	2000
High-density Polyethylene	20	1400
Nylon-6	150	25–40
Polyvinylidene chloride	0.4–1	<1
Ethylene-vinyl alcohol copolymer	20–70	0.1–1
PBS	-	1200
PLA	325	550
Ecoflex (BASF)	170	1400
Ecoflex/PPC/PBS triple-coextruded film	5	9.3
Ecoflex/PPC/LDPE triple-coextruded film	5.3	9.5

**Table 6:** Modified materials and enhanced performance.

No.	Materials	Preparation Method ^(a),(b)^	Amount Added (wt%)	Performance Enhancement						Reference
				Mechanical Behavior		Thermal Properties				
				Tensile Strength/MPa	Elongation at Break/%	Tg/°C	T_d−5%_/°C ^(c)^	T_d−10%_/°C ^(d)^	T_max_/°C^(e)^	
1	Carbon fiber	M	0–20	-	-	42	-	-	-	[71]
2	Graphene/Si hybrids	M	0–5	35.5 ± 1.3	36.7 ± 1.5	34.2	289.5	-	-	[72]
3	Elokite nanotubes	M	0–10	22.6	-	-	285.1	311.3	311.3	[73]
4	Montmorillonite	S	0~10	-	-	-	-	280 °C	-	
5	Laponite	S	0–10	-	-	-	-	250	-	[74]
6	Activated white clay	S	0–2	36.8 ± 1.7	92 ± 16	32.7	260	-	276	[75]
7	Boehmite	M	0–20	37.83	lower	-	399.9	410	439.4	[76]
8	-modified sepiolite	S	0–10	25.6	216	35.8	288.7	-	322.9	[77]
9	Nanosilica	S	-	15	498	37	238	-	-	[78]
10	Calcium carbonate	M	0–20	36.6	-	-	256	-	292	[79]
11	Aluminum hydroxide	M	0–20	31.54	-	-	-	-	-	[80]

^(a)^ M: melt blending method. ^(b)^ S: solution comingling method. ^(c)^ T_d−5_%: thermal decomposition temperature at 5 wt% loss. ^(d)^ T_d−10_%: thermal decomposition temperature at 10 wt% loss. ^(e)^ T_max_: maximum thermal decomposition temperature.

## Data Availability

Not applicable.

## Appendix A

**Table A1:** Abbreviated table.

First Appearance	Shorthand	Full Name	First Appearance	Shorthand	Full Name
1	CO_2_	Carbon dioxide	11	MMT	Montmorillonite
2	CH_4_	Methane	11	AC	Activated white clay
2	pH	Hydrogen ion concentration	11	ATH	Aluminum hydroxide
2	TPS	Thermoplastic starch	12	CS	Chitosan
2	PHA	Polyhydroxyalkanoate	12	NCC	Nanocellulose
2	PGA	Polyglutamic acid	13	AL	Alkaline lignin
2	PBS	Polybutylene succinate	13	BLF	Black liquor lignin
2	PBAT	Polybutyleneadipate-co-terephthalate	13	HBL	Hydroxy black liquor lignin
2	PCL	Polycaprolactone	13	PPCMA	Maleic anhydride grafted PPC
2	PPC	Polypropylene carbonate	13	TPOS	Thermoplastic starch oxide
2	PLA	Polylactic acid	13	DL-TPOS	Aluminate pretreated starch oxide
2	PE	Polyethylene	14	CSS NPs	Core-shell starch nanoparticles
2	PP	Polypropylene	14	PMA	Poly (methyl acrylate)
3	PBSA	Polybutylene succinate-co-butylene adipate	14	PHBV	β-hydroxyvalerate copolymer
4	PVA	Polyvinyl alcohol	14	WP	Wool powder
4	PO	Propylene oxide	14	LCHBP	Long-chain hyperbranched polymer
6	T_g_	Glass transition temperature	16	CA	Cellulose acetate
6	T_m_	Melting temperature	17	HAP	Hydroxyapatite
